# Plastic Traits of an Exotic Grass Contribute to Its Abundance but Are Not Always Favourable

**DOI:** 10.1371/journal.pone.0035870

**Published:** 2012-04-20

**Authors:** Jennifer Firn, Suzanne M. Prober, Yvonne M. Buckley

**Affiliations:** 1 School of Earth, Environment and Biological Sciences, Queensland University of Technology, Brisbane, Queensland, Australia; 2 Ecosystem Sciences, CSIRO, Wembley, Western Australia, Australia; 3 School of Biological Sciences, The University of Queensland, St. Lucia, Queensland, Australia; 4 Ecosystem Sciences CSIRO, Dutton Park, Queensland, Australia; Duke University, United States of America

## Abstract

In herbaceous ecosystems worldwide, biodiversity has been negatively impacted by changed grazing regimes and nutrient enrichment. Altered disturbance regimes are thought to favour invasive species that have a high phenotypic plasticity, although most studies measure plasticity under controlled conditions in the greenhouse and then assume plasticity is an advantage in the field. Here, we compare trait plasticity between three co-occurring, C_4_ perennial grass species, an invader *Eragrostis curvula*, and natives *Eragrostis sororia* and *Aristida personata* to grazing and fertilizer in a three-year field trial. We measured abundances and several leaf traits known to correlate with strategies used by plants to fix carbon and acquire resources, i.e. specific leaf area (SLA), leaf dry matter content (LDMC), leaf nutrient concentrations (N, C∶N, P), assimilation rates (*Amax*) and photosynthetic nitrogen use efficiency (PNUE). In the control treatment (grazed only), trait values for SLA, leaf C∶N ratios, *Amax* and PNUE differed significantly between the three grass species. When trait values were compared across treatments, *E. curvula* showed higher trait plasticity than the native grasses, and this correlated with an increase in abundance across all but the grazed/fertilized treatment. The native grasses showed little trait plasticity in response to the treatments. *Aristida personata* decreased significantly in the treatments where *E. curvula* increased, and *E. sororia* abundance increased possibly due to increased rainfall and not in response to treatments or invader abundance. Overall, we found that plasticity did not favour an increase in abundance of *E. curvula* under the grazed/fertilized treatment likely because leaf nutrient contents increased and subsequently its' palatability to consumers. *E. curvula* also displayed a higher resource use efficiency than the native grasses. These findings suggest resource conditions and disturbance regimes can be manipulated to disadvantage the success of even plastic exotic species.

## Introduction

Exotic plant species can establish and dominate sites despite lacking evolutionary familiarity with local conditions and having small founder populations (i.e. the invasion paradox [1], [2]). Substantial evidence suggests disturbances such as changed grazing regimes and nutrient addition increase opportunities for invasive species to establish [3], [4], [5], [6], [7], [8], particularly if disturbances are novel to an ecosystem [9], [10]. Disturbance favours the growth and survival of some species over others depending on the characteristics of the disturbance itself including the type, frequency, duration and intensity [11], but also on the traits of species present [12], [13]. Despite extensive research, evidence for a generic set of traits that favour exotic over native species remains inconclusive [14], [15], [16], [17].

Evidence suggests invasive species tend to display traits of fast growing species that are resource acquisition specialists and native species tend to display traits of slow-growing species that are conservation specialists [18], [19], [20], [21], [22]. The leaf economic spectrum proposes a fundamental trade-off in the traits held by fast- and slow-growing plant species [23], [24], [25]. Fast growing species, better at resource capture, tend to dominate disturbed ecosystems where resource availability is not limited. These fast growing species have generally higher specific leaf area (SLA, mm^2^/mg, fresh leaf area/oven-dry mass), lower leaf dry matter content (LDMC, mg/g, oven dry mass/water-saturated fresh mass), higher nutrient contents and higher rates of assimilation (*Amax*) [23], [24], [25]. Slower growing plant species generally occupying low resource and less disturbed sites are better at resource conservation and to tend to hold opposite traits—lower SLAs, higher LDMCs, lower nutrient contents and lower rates of *Amax* [23], [24], [25]. Studies comparing the leaf traits of exotics and natives have consistently found evidence for this trade-off, with exotics showing better resource acquisition strategies and natives better resource conservation strategies [26]. However, recent findings by Leishman et al. [27] suggest that exotic and native plant species can hold similar strategies for capturing resources, with exotic and native species at disturbed sites possessing similar traits, but different traits to natives at pristine sites.

To date, most studies investigating plant traits focused on differences between species (interspecific variability) and across sites affected by different disturbances and environmental conditions, but recent research has highlighted the importance of intraspecific variability in traits or phenotypic plasticity [28], [29]. Evidence suggests that invasive exotic species display higher phenotypic plasticity than natives—the potential of each individual genotype to produce different traits/phenotypes in response to disturbance and fluctuating environmental conditions [14], [30], [31]. This capacity to change morphological or physiological traits may allow genotypes of a species to thrive across a wider range of environmental conditions (genotype-level plasticity), and/or allow individuals within a population to thrive at sites during and after disturbance or resource pulses (species-level plasticity) [14], [17], [30], [32], [33].

Invasive species have shown higher trait plasticity in response to increased resources, e.g soil nutrients and water, in comparison to phylogenetically-related non-invasive species from high resource environments [34], [35], and phylogenetically-related native species from low resource environments [33]. Although a recent greenhouse study comparing 20 phylogenetically-related invasive and native trees and shrubs found similar levels of trait plasticity in response to nutrient and light treatments, but enhanced performance by invasive species measured as mean trait values [36]. Studies have also shown individuals of the same species sampled from both introduced and native sites have a higher trait plasticity at introduced sites [37]. However, studies measuring trait plasticity have generally grown species over short-periods of time under controlled greenhouse conditions. Adults growing in the field may display different morphological and physiological traits when subjected to a wider range of resource conditions and biotic interactions in comparison to controlled greenhouse experiments [38].

Here, we use a factorial field trial to compare trait plasticity between an invasive exotic grass (*Eragrostis curvula* (Schrad.) Nees, hereafter lovegrass), and two native grasses (*Aristida personata* Henrard, hereafter purple wiregrass, and *Eragrostis sororia* Domin, hereafter woodlands lovegrass). Our study is unique as we measure how traits of key species in a community change in response to treatments, and measure these changes under ‘realistic conditions’ to increase the reliability of the results for explaining invasion success. We measured how grazing and fertilizer addition treatments altered abundances and several leaf traits. We hypothesised that under the existing site conditions (grazing) that the invader would display traits consistent with faster growth than the natives. We also hypothesised that under different experimental treatments the invader would exhibit higher phenotypic plasticity than the natives, evidenced by predictable changes in traits based on trends identified in the leaf economic spectrum. We then relate these results to differences in abundance of all three species between the treatments. This invasion scenario is a model system to compare plasticity because these species share life-history traits, co-exist at the same site, and native woodlands lovegrass is a congener of the invader lovegrass (Table 1).

**Table 1:** General characteristics of the invasive exotic lovegrass, and the native grasses purple wiregrass and woodlands lovegrass.

Characteristics	*Eragrostis curvula* lovegrass, Exotic grass	*Aristida personata* purple wiregrass Native grass	*Eragrostis sororia* woodlands lovegrass Native grass
Mean abundance at site (± S.E.) at time 0	47.56%±3.98	22.66±5.12	1.61%±0.67
Growth Habit	Tufted perennial	Tufted perennial	Tufted perennial
Photosynthetic Pathway	C_4_	C_4_	C_4_
Height	Up to 120 cm	Up to 120 cm	Up to 70 cm
Growth season	Summer	Summer	Summer
Flowering time	Spring to Autumn	Summer to Autumn	Summer
Palatability to livestock	Low	Low	Moderate
Native continental distribution	Africa	Australia	Australia

[61], [62], [63], [64], [65].

## Results

**Figure 1:**
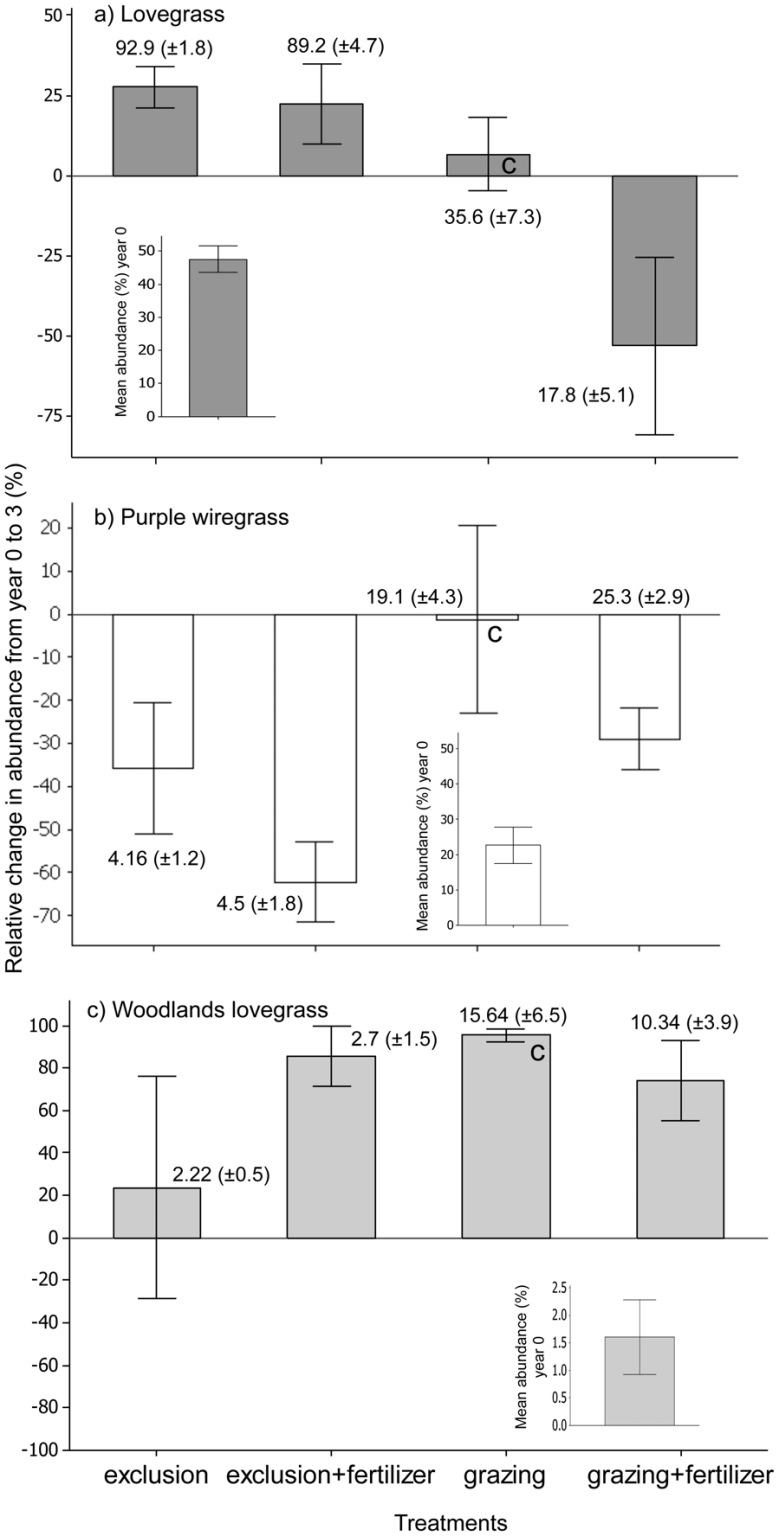
Relative change in abundance of each species from measurements taken prior to the start of the field trial and measurements taken again after three years of treatments (calculated as ((abundance_T3_-abundance_T0_)/abundance_T0_)×100). The insets show the mean abundance values (± S.E.) at time 0 and the values shown next to each bar are the mean abundance values (± S.E.) after three years. **C** indicates the control treatment grazing/no fertilizer.

After three years of treatments, the abundance of all species was significantly correlated with abundance prior to the start of the treatments, time 0 (Table 2). Lovegrass (exotic) abundance was best explained by the additive effects of grazing and fertilizer, but not the interaction (Table 2 a). Lovegrass abundance increased across all treatments except the grazed/fertilized treatment where its abundance decreased (Fig. 1 a, 53.11%±27.80 reduction in comparison to time 0). Grazing treatments had the strongest effect (*F*_1, 2_ = 139.91, *P*<0.008); but fertilizer treatments also had a significant effect (*F*_1, 54_ = 6.14, *P*<0.02). Purple wiregrass abundance was best explained by the effect of the grazing treatment (*F*_1, 2_ = 44.51, *P*<0.025). After three years, the abundance of purple wiregrass was reduced across all treatments, but most significantly in the grazing exclusion treatments (>35% decrease, Fig. 1 b), which was also the treatment where lovegrass abundance increased the most (>20% increase Fig. 1a). Woodlands lovegrass was low in abundance pre-treatment (Table 1), and increased across all treatments when compared to its abundance at year 0. It increased in abundance in the grazing exclusion treatments to more than 2% and in the grazed treatments to more than 10% (Fig. 1 c), but the difference between treatments was not significant.

**Table 2:** Results from an ANOVA conducted to assess the significance of the fixed effects for LMEMs of abundance (arc-sine transformed) in year 3, with a fixed effects structure of grazing and fertilizer treatments and a co-variate of abundance in time 0, and a random effects structure of block/plot.

abundance_time 3_	Fixed effects	F values (df as subscript), *P* value
a) Lovegrass	**grazing**	***F*** **_1, 2_ = 139.91, ** ***P*** **<0.008**
	**fertilizer**	***F*** **_1, 54_ = 6.14, ** ***P*** **<0.02**
	**abundance_time 0_**	***F*** **_1, 54_ = 48.79, ** ***P*** **<0.0002**
	grazing×fertilizer	*F*_1, 54_ = 1.96, *P*<0.20
	grazing×abundance_time 0_	*F*_1, 54_ = 1.98, *P*<0.20
	fertilizer×abundance_time 0_	*F*_1, 54_ = 0.38, *P*<0.60
	grazing×fertilizer×abundance_time 0_	*F*_1, 54_ = 0.26, *P*<0.65
b) Purple wiregrass	**grazing**	***F*** **_1, 2_ = 44.51, ** ***P*** **<0.025**
	fertilizer	*F*_1, 54_ = 0.25, *P*<0.70
	**abundance_time 0_**	***F*** **_1, 54_ = 33.25, ** ***P*** **<0.002**
	grazing×fertilizer	*F*_1, 54_ = 0.02, *P*<0.90
	grazing×abundance_time 0_	*F*_1, 54_ = 0.16, *P*<0.70
	fertilizer×abundance_time 0_	*F*_1, 54_ = 1.16, *P*<0.30
	grazing×fertilizer×abundance_time 0_	*F*_1, 54_ = 0.31, *P*<0.60
c) Woodlands lovegrass	grazing	*F*_1, 2_ = 4.18, *P*<0.20
	fertilizer	*F*_1, 32_ = 4.18, *P*<0.20
	**abundance_time 0_**	***F*** **_1, 32_ = 8.00, ** ***P*** **<0.04**
	grazing×fertilizer	*F*_1, 32_ = 1.18, *P*<0.30
	grazing×abundance_time 0_	*F*_1, 32_ = 2.29, *P*<0.20
	fertilizer×abundance_time 0_	*F*_1, 32_ = 0.91, *P*<0.40
	grazing×fertilizer×abundance_time 0_	*F*_1, 32_ = 0.13, *P*<0.80

In year 3, the availability of soil nutrients also varied significantly depending on the treatments (Fig. S1 and Table S1). Soil nitrate (NO_3_) levels varied marginally by the interaction of grazing and fertilizer treatments (*F*_1, 58_ = 3.00, *P*<0.09, Fig. 1 a), although overall nitrate levels were higher in the grazing exclusion treatments and highest in the grazing exclusion and unfertilized treatment. This decreasing trend in soil nitrate levels, despite the application of fertilizer, is likely reflective of increased leaching and/or use by plants and soil fauna. Soil ammonium (NH_4_) levels did not vary significantly between the treatments (Fig. S1b and Table S1b). Soil phosphate (PO4) levels increased significantly with the grazing treatments (*F*_1, 2_ = 30.53, *P*<0.03, Fig. 1 c) and the fertilizer treatments (*F*_1, 58_ = 11.67, *P*<0.001, Fig. 1 c), but not the interaction.

### Traits differed between species in the control treatment

**Figure 2:**
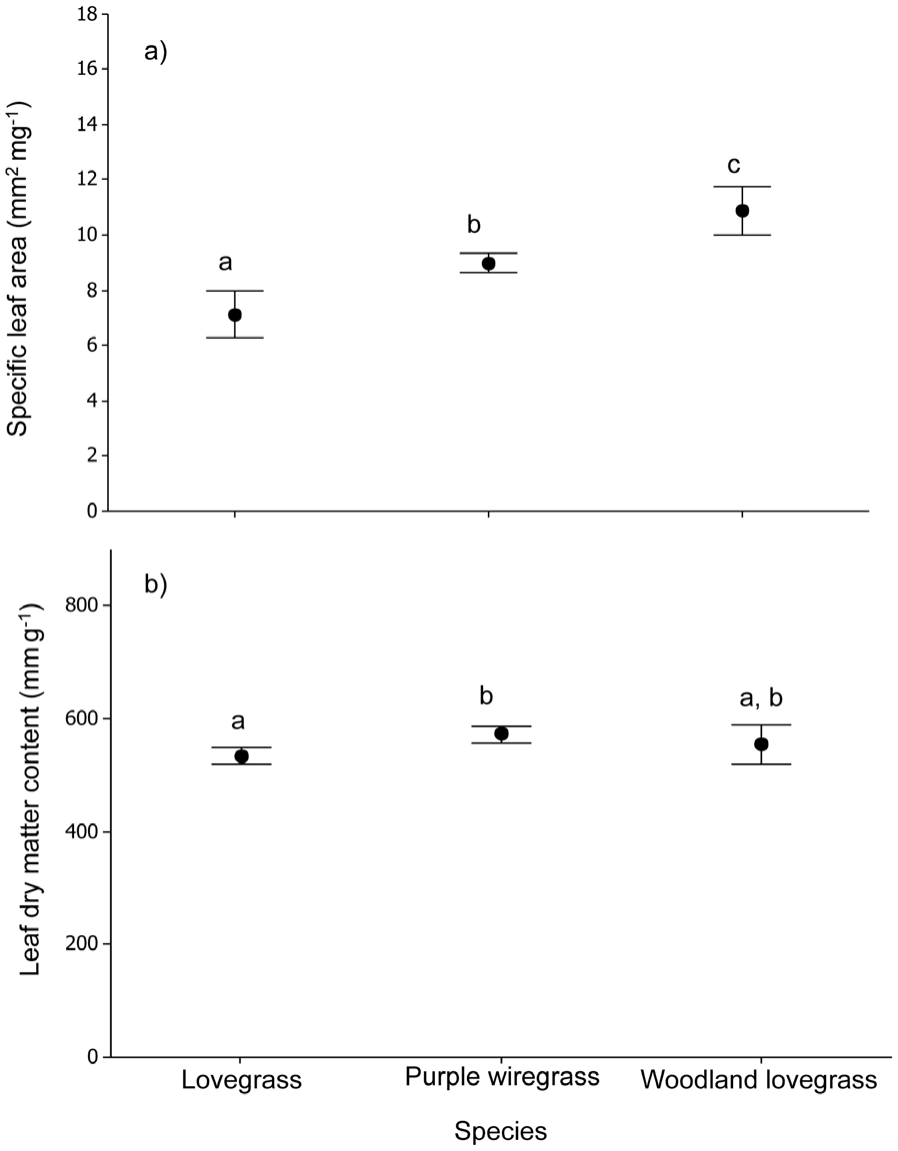
Comparison of mean trait values between species at the site level. Mean LDMC and SLA values (± S.E.) for each species for the grazing only treatment, which was the original disturbance at this site and therefore represents a control. Different letters indicate means are significantly different at p<0.05.

**Figure 3:**
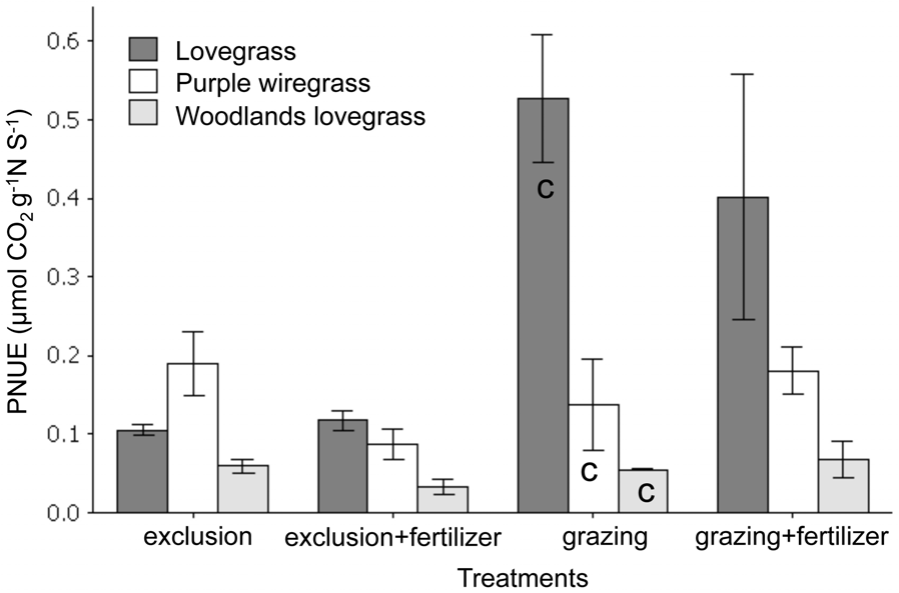
Mean photosynthetic nitrogen use efficiency (± SE) for each species depending on the grazing and fertilizer treatments. **C** indicates the control treatment grazing/no fertilizer.

**Figure 4 pone-0035870-g004:**
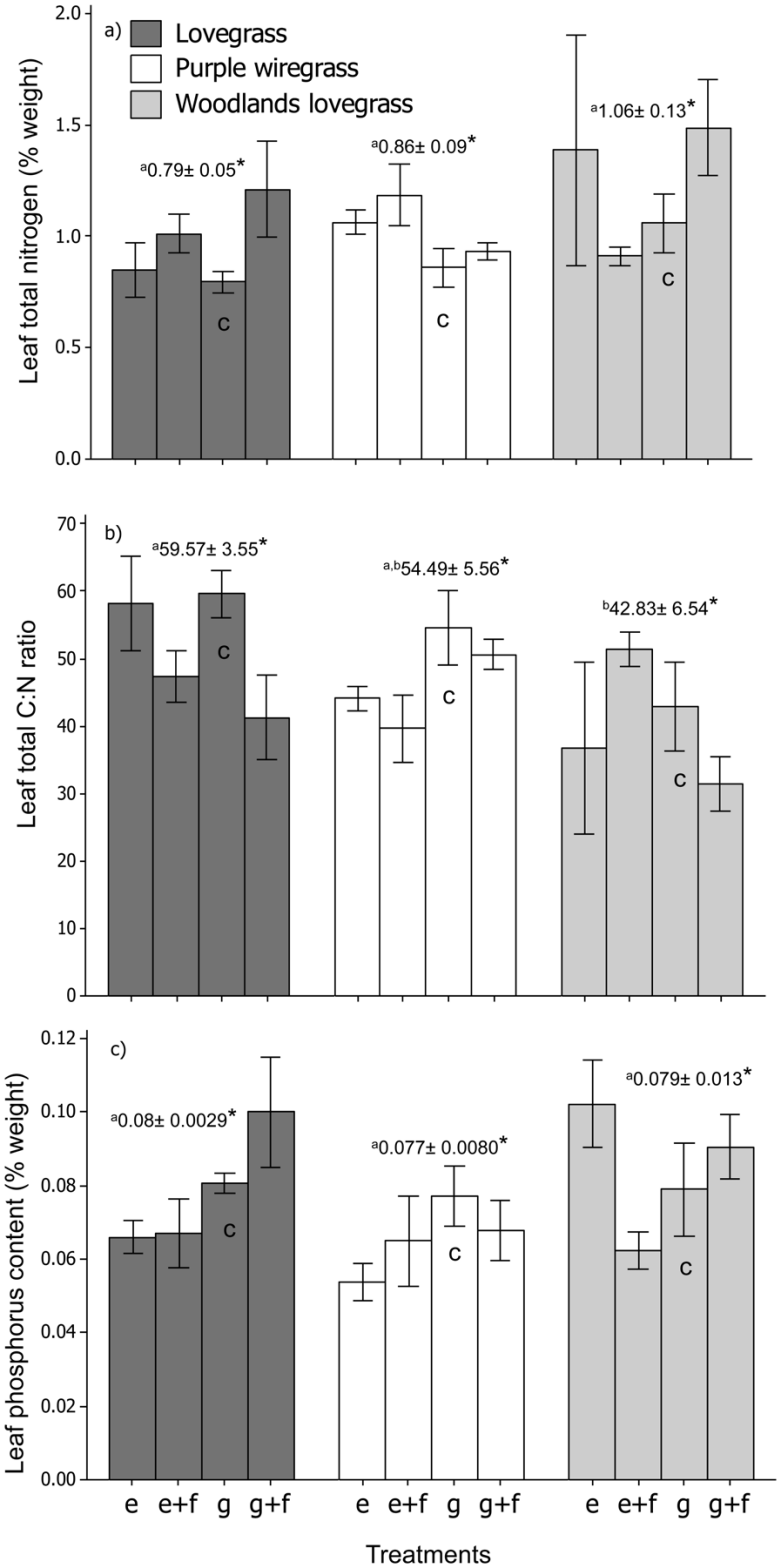
Mean leaf nutrient concentrations (± SE) for each species depending on the grazing and fertilizer treatments. Panel a) shows leaf total nitrogen concentration (% weight), b) leaf carbon to nitrogen ratios and c) leaf phosphorus concentration (% weight). Values shown in each panel are the mean leaf nutrient concentrations (± SE) for each species at the site regardless of treatment. Different letters indicate means are significantly different at p<0.05. **C** indicates control treatment, grazing/no fertilizer.

In the control treatment (grazed/no fertilizer), mean LDMC values did not vary significantly between species (*F*_2, 60_ = 0.89, *P*<0.50, Fig. 2 a). Mean SLA values differed marginally between species, but contrary to expectations, with lovegrass showing a lower mean SLA value than Purple wiregrass (*F*_2, 60_ = 2.69, *P*<0.08, Fig. 2 b), a trait indicative of a slower growing species. In agreement with expectations that lovegrass would display characteristics of a faster growing species under the grazing treatment (control), lovegrass had a significantly higher assimilation rate (*Amax*; *F*_2, 30_ = 10.1, *P*<0.002, Fig. S2 “grazing") and photosynthetic nitrogen use efficiency (PNUE = *Amax*/leaf nitrogen; *F*_2, 30_ = 8.78, *P*<0.001, Fig. 3 “grazing") than the two native grasses. Leaf nutrient concentrations in the control treatment did not vary significantly between species, except in the case of Leaf C∶N ratios where lovegrass showed a significantly higher ratio than woodlands lovegrass (*F*_2, 30_ = 3.13, *P*<0.05, Fig. 4 b), again contrary to expectations as this is a trait indicative of a slower growing species.

### Trait plasticity in response to the treatments differed amongst species

**Figure 5:**
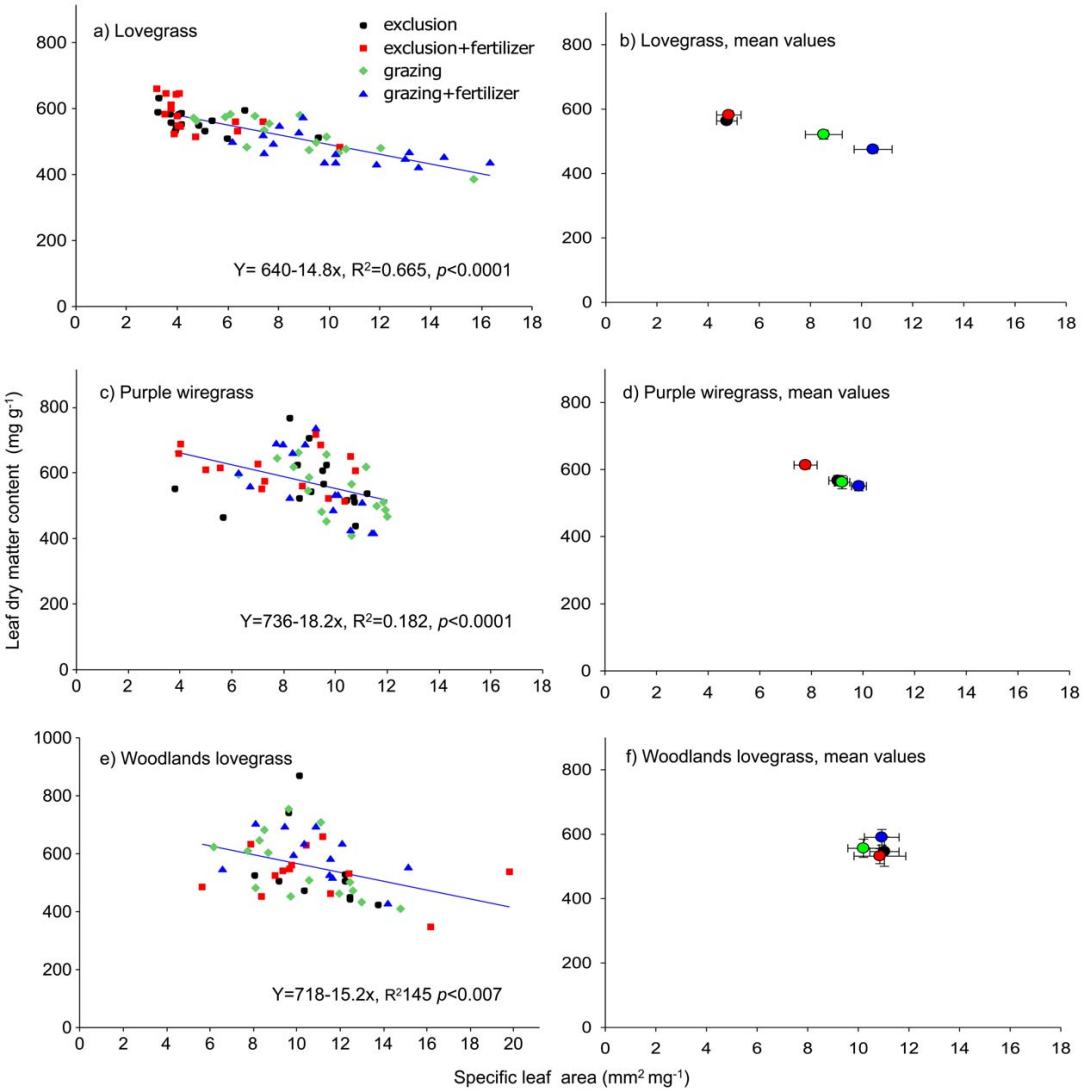
Correlations between LDMC and SLA values for each species depending on the four grazing and fertilizer treatments. Panel a), c) and e) show the mean LDMC and SLA values for each species collected from each plot and b), d), f) show the mean values for LDMC and SLA (±) for each treatment.

The traits of lovegrass varied predictably with the treatments, with adult individuals showing significant differences in LDMC, SLA, PNUE, *Amax* and leaf nutrients. The traits of purple wiregrass also changed with the nutrient treatments, but woodlands lovegrass showed little change (Fig. 3–5, Fig. S2, Table 3 – 5 and Table S1). Differences in LDMC and SLA values for lovegrass were best explained by the interaction of grazing and fertilizer treatments (LDMC: *F*_1, 58_ = 10.10, *P*<0.002 and SLA: *F*_1, 58_ = 3.89, *P*<0.05, Table 3). In agreement with expectations, LDMC decreased and SLA increased for lovegrass with increasing amounts of disturbance from grazing exclusion treatments to the grazed/fertilized treatments (Fig. 5 a and b). The highest LDMC and lowest SLA values were found in both grazing exclusion treatments, whereas the lowest LDMC and highest SLA values were shown in the grazed/fertilized treatments. For purple wiregrass, differences in LDMC were not explained by the grazing or fertilizer treatments, whereas differences in SLA were explained by fertilizer treatments (*F*_1, 58_ = 4.25, *P*<0.05, Table 3).

**Table 3:** Results from an ANOVA of LMEMs of leaf dry matter content (LDMC) and specific leaf area (SLA) for each of the grass species, with a fixed effects structure of grazing and fertilizer treatments and a random effects structure of block/plot.

Species & response variable	Fixed effects	*F* values (dfs as subscript), *P* values
a) Lovegrass LDMC (mg g^−1^)	grazing	*F*_1, 2_ = 5.60, *P*<0.20
	fertilizer	*F*_1, 58_ = 2.58, *P*<0.10
	**grazing×fertilizer**	***F*** **_1, 58_ = 10.10, ** ***P*** **<0.002**
SLA (mm^2^ mg^−1^)	grazing	*F*_1, 2_ = 8.85, *P*<0.10
	**fertilizer**	***F*** **_1, 58_ = 4.55, ** ***P*** **<0.04**
	**grazing×fertilizer**	***F*** **_1, 58_ = 3.89, ** ***P*** **<0.05**
b) Purple wiregrass LDMC (mg g^−1^)	grazing	*F*_1, 2_ = 0.40, *P*<0.60
	fertilizer	*F*_1, 58_ = 1.91, *P*<0.20
	grazing×fertilizer	*F*_1, 58_ = 1.00, *P*<0.30
SLA (mm^2^ mg^−1^)	grazing	*F*_1, 2_ = 0.64, *P*<0.50
	**fertilizer**	***F*** **_1, 58_ = 4.25, ** ***P*** **<0.05**
	grazing×fertilizer	*F*_1, 58_ = 0.28, *P*<0.60
c) Woodlands lovegrass LDMC (mg g^−1^)	grazing	*F*_1, 2_ = 0.08, *P*<0.80
	fertilizer	*F*_1, 58_ = 0.17, *P*<0.70
	grazing×fertilizer	*F*_1, 58_ = 0.51, *P*<0.50
SLA (mm^2^ mg^−1^)	grazing	*F*_1, 2_ = 0.10, *P*<0.80
	fertilizer	*F*_1, 58_ = 0.54, *P*<0.50
	grazing×fertilizer	*F*_1, 58_ = 0.35, *P*<0.60

**Table 4:** For each of the three grass species, treatments that significantly predicted differences in total leaf nitrogen concentration, leaf carbon to nitrogen ratio and total leaf phosphorus concentration.

	Predictor variables	*F* values (df as subscript), *P* value
a) Lovegrass leaves		
Total nitrogen (% weight)	Fertilizer treatment	*F*_1_ = 4.67, *P*<0.06
C∶N ratio	Fertilizer treatment	*F*_1_ = 7.26, *P*<0.02
Total phosphorus (% weight)	Grazing treatment	*F*_1_ = 6.16, *P*<0.03
b) Purple wiregrass leaves		
Total nitrogen (% weight)	Grazing treatment	*F*_1_ = 5.33, *P*<0.08
C∶N ratio	Grazing treatment	*F*_1_ = 7.17, *P*<0.06
Total phosphorus (% weight)	NS	
d) Woodlands lovegrass leaves		
Total nitrogen (% weight)	NS	
C∶N ratio	Grazing treatment	*F*_1_ = 7.82, *P*<0.06
Total phosphorus (% weight)	Grazing treatment	*F*_1_ = 9.33, *P*<0.02

**Table 5:** Summary of the traits of each grass species that showed a plastic response to the treatments according to expectations “√", contrary to expectations “X" or traits that did not change in response to the treatments “-".

Trait	*Eragrostis curvula* lovegrass, Exotic grass	*Aristida personata* purple wiregrass Native grass	*Eragrostis sororia* woodlands lovegrass, Native grass
LDMC (mg g^−1^)	✓ (grazing & fertilizer)	-	-
SLA (mm^2^ mg^−1^)	✓ (grazing & fertilizer)	✓ (fertilizer)	-
*Amax* (µmol co_2_ g^−1^ s^−1^)	✓ (grazing)	-	-
PNUE (µmol co_2_ g^−1^N s^−1^)	✓ (grazing)	-	-
Leaf total Nitrogen (%)	✓ (fertilizer)	X (grazing)	-
Leaf total C∶N	✓ (fertilizer)	X (grazing)	X (grazing)
Leaf Phosphorus (%)	✓ (grazing)	X (grazing)	X (grazing)

PNUE varied depending on the interaction between species and treatments (*F*_6, 42_ = 2.38, *P*<0.05). Lovegrass showed a three-fold increase in PNUE between the grazed and exclusion treatments (Fig. 3). Woodlands lovegrass overall had a lower PNUE than the other grasses, but rates did not vary between treatments (Fig. 3). Purple wiregrass had a higher PNUE rate than woodlands lovegrass, but did not show a significant difference between treatments (Fig. 3). *Amax* varied similarly to PNUE depending on the interaction between species and treatments (*F*_6, 42_ = 2.84, *P*<0.02, Fig. S2).

Differences in total nitrogen concentration were marginally significant and C∶N ratio were significant for lovegrass leaves collected from fertilized and unfertilized treatments (Fig. 4 a and b, Table 4 a and Table S2 a). Lovegrass leaves showed a marginally significant increase in leaf nitrogen concentration in the fertilized plots, with the highest increase occurring in the grazed/fertilized treatment (Fig. 4 a). Consistent with this increase in N, leaf C∶N ratios for lovegrass decreased when fertilizer was added (Fig. 4 b and Table S2 a). The total phosphorus concentration of lovegrass leaves varied significantly with the grazing treatment, with the highest phosphorus concentration occurring in treatments where grazing was maintained (Fig. 4 c, Table 4 a and Table S2 a). Total leaf phosphorus for purple wiregrass did not vary significantly between treatments, but total nitrogen and C∶N ratios differed marginally with the grazing treatment (Table 4 b and Table S2 b). The total nitrogen concentration of purple wiregrass leaves was lower in the grazed treatment, and C∶N ratios higher in the grazed treatment for purple wiregrass. The nitrogen concentration of woodlands lovegrass leaves did not vary significantly, but did vary depending on the grazing treatments for both C∶N ratios and total phosphorus concentration (Table 4 c and Table S2 c). In both cases, woodlands lovegrass leaves, collected from the exclusion treatment, had the highest C∶N ratios and the highest total phosphorus concentration (Fig. 4 c).

Table 5 summarises the response of each trait to the grazing and fertilizer treatments for each of the three grass species and indicates whether the change followed or was contrary to expectations.

## Discussion

Overall we found the invasive exotic grass displayed higher trait plasticity in response to the treatments than the two native grasses (Table 5). Lovegrass changed its traits according to predictions based on trends from the Leaf Economic Spectrum for all six traits, compared with only one trait for the native grasses [23]. A recent meta-analysis comparing 75 invasive/non-invasive pairs of plant species found invaders were more plastic in their response to increased resource availability than non-invaders, but plasticity was only a fitness advantage for the invasive species when resource conditions were high [39]. Increased resource availability is widely agreed to promote invasion [40], [41], [42]. Because most studies in this meta-analysis were pot trials growing plants in the absence of competition and other biotic interactions such as grazing, it is difficult to extrapolate these findings to field conditions [38]. We found the plastic response of Lovegrass was not an advantage in the field when resources are high as increased soil nutrients, led to increased resource uptake by the exotic but also increased selective grazing pressure.

Using a three year field study, we found increased nutrients coupled with grazing decreased the abundance of the exotic, a trend also measured in the first two years of the study and published in Firn et al. [43]. Under these conditions lovegrass leaves increased in SLA, decreased in LDMC and increased in leaf total nitrogen and phosphorus concentration in response to fertilizer, but this response likely also increased its palatability to grazing livestock. An alternative explanation for these results to trait plasticity may be increased genetic diversity prior to the start of the experiment within the lovegrass population, and the treatments led to differential survival or ‘filtering’ of phenotypes better adapted to the different experimental conditions. This explanation is, however, unlikely as the grasses are long-lived perennials and we were careful to measure traits from large mature tussocks. Also, if genetic diversity were the explanation, genotypes in the experimental treatments would likely be subsets of those in the control; therefore, we would have expected higher trait variation in the control treatment (grazing only).

The leaf traits of lovegrass also changed in the exclusion treatments showing lower SLA and higher LDMC suggesting it has at least a comparable capacity to conserve resources as the native grasses. Using a greenhouse study, Funk [33] compared the response of several related exotic and native species from resource limiting environments and also found exotics were equally or more efficient at resource conservation. Lovegrass and both native species showed similar mean traits under the control treatment of grazing only, except the exotic had a lower SLA (indicative of a slower growing species) than purple wiregrass and a higher PNUE and *Amax* than both native species. This result suggests lovegrass is more efficient at resource capture than the native species. A study comparing traits of exotics to native species in the same region of Australia, found several C_4_ exotic grass species (including lovegrass), had higher LDMC than native species [44], similarly suggesting successful exotic grasses in this region may be resource conservation specialists [44].

We also found evidence that lovegrass has a higher resource use efficiency (RUE, carbon assimilation per unit of resource, measured as PNUE) than the native grasses. Funk and Vitousek [45] compared RUE between related and co-occurring exotic and native species within Hawaii, and also found exotics had a higher RUE. PNUE increased more than three-fold in the grazed versus exclusion treatments. Leaf C∶N ratios decreased in the treatments that were fertilized, but this same response was not shown by the native grasses. Higher RUE would be an advantage at the field site, as rainfall is highly variable and soil nutrients low.

Lovegrass was the dominant species at the site in year 0 and displayed the highest plasticity in response to grazing and fertilizer after three years of treatment, and this plasticity correlated with changes in its abundance in the short-term. Grime's mass ratio hypothesis [46] describes dominant species as having the highest impact on ecosystem functions. Dominant species may then be the most plastic in response to changed conditions. While intermediate/subordinate species abundance may be most influenced by the abundance of the dominant species and transient species (a species whose abundance fluctuates depending on resources) abundance responsive to environmental fluctuations [46].

Purple wiregrass, a subordinate species, was reduced in abundance across the treatments with the highest reductions occurring where grazing was excluded. Purple wiregrass did show some trait plasticity. Although it was the least disturbed treatments where purple wiregrass showed some plasticity, including an increased SLA in exclusion/fertilised treatment and an increased leaf nitrogen concentration in the exclusion treatments. In accordance with Grime's mass ratio hypothesis, increased abundance of lovegrass (>85%) in the exclusion treatments may account for the significant reduction of purple wiregrass abundance (reduced by >35%).

Woodlands lovegrass increased in abundance across the treatments. Although related to lovegrass, woodlands lovegrass did not show similar trait plasticity. This finding suggests that the increased abundance of woodlands lovegrass may be driven by other factors such as increased rainfall in year 3 of the study. Mean rainfall in year 0 was 215 mm, which was lower than the local 20 year average of 600 mm [47]; while mean rainfall in year 3 was higher than the local average at 652 mm.

At disturbed sites, invasive exotic species may be successful because of traits that allow quick growth in response to increased resource conditions [40], [41], but in generally low resource environment these species likely also need traits that temper growth to survive lulls between resource pulses [48]. Pursuit of a tangible set of generic traits that distinguish exotics from natives may not be plausible or meaningful [49]; instead, we suggest the pursuit should focus on plasticity, as this may be the trait that leads to characteristically dominant plant species whether native or exotic. Lovegrass may have replaced a more plastic and characteristically dominant native species, and future studies should compare invasive and native species that are all generally considered to hold a similar hierarchical role in a community (i.e. compare dominants to dominants, subordinates to subordinates and rare to rare).

Overall, our results show that plasticity at the species level, however, does not necessarily equate to a ‘super invader’; instead, if plasticity of an undesirable species is understood, biotic interactions and resource availability can be manipulated to limit abundance. Exotic species with high species-level phenotypic plasticity may then be vulnerable to changed resource conditions as a direct result of this plasticity.

## Materials and Methods

### Study species

Lovegrass was introduced into Australia in the early 1900s for pasture improvement and soil conservation [50], and is now found in every Australian state, spreading into many regions where it was never intentionally introduced [51]. The increased dominance of lovegrass poses a significant threat to native biodiversity because of its ability to dominant communities, and the sustainability of production in farming communities because it is not palatable (low nutrients and crude protein content) to grazing livestock in the low productivity regions where it is spreading, and difficult to control [51].

In June 2006, we established a large field trial on a private cattle grazing property in the Millmerran region of south-western Queensland, Australia [43]. The field site had been grazed by cattle with a low stocking rate since 1980 and has never been cultivated or fertilized. Lovegrass was first identified on the property in 1998 by the landholder. Average rainfall of this area is 600 mm p.a. [47] with two-thirds of the rain occurring during the summer months from October to April. The soil is a yellow sodosol derived from sandstone, which is characteristically low in nutrients, slightly acidic (ranging from pH 4.8 to 5.9), low in water holding capacity, and highly susceptible to compaction [47].

### Data collection and sampling design

In this experiment, we measured traits and abundances from a subset of treatments and plots from a larger field trial with a randomized split-plot design [43]. In June 2006, four large blocks (35×40 m) were established randomly in a pasture dominated by lovegrass. Two blocks were fenced to exclude grazing by cattle and limit access by other native and exotic gazers (e.g. kangaroos, wallabies, hares and rabbits). The other two blocks were left open to grazing. In each block, we established 48 plots (each was 9 m^2^ in size with an additional 4 m^2^ buffer between each plot). In this study, we sampled 16 plots in each block, 8 fertilized plots and 8 unfertilized plots. We applied a slow-release fertilizer to half of the plots in a pellet form (N 21.6%, P 1.1%, K 4.1%) at a low application rate of 2 kg/ha at the start of each growing season from 2006 to 2009, which can begin anytime between October and December depending on rainfall. In this experiment, the grazed/-unfertilized treatment is considered the control treatment, because this was the disturbance acting on the site prior to the start of the experiment. Firn *et al.* (2010) contains species abundance results from 2006 to 2008.

In December 2009, prior to applying the fourth year of treatments, we measured species abundance and leaf traits (specific leaf area, leaf dry matter content, *Amax* and leaf nitrogen, phosphorus, and carbon to nitrogen levels) from the grazing and fertilizer treatment combinations. The abundances of all species were recorded within each plot in the central 9 m^2^ section using the point-intercept method (modified from [52]). A 4 mm dowel was placed vertically on set points along a grid of 100 points. Relative abundance was measured by identifying and counting each leaf, stem and inflorescence that touched the dowel at each point along the grid.

To measure SLA, LDMC, LN traits, we collected five young but fully expanded leaves from three mature individuals of each species within each of the 64 plots, using the standardised protocols detailed by Cornelissen et al. [53] and the rehydration methods proposed by Garnier et al. [54]. Because of the low abundance of woodlands lovegrass, we did not find individuals of this species in all plots, but we were able to collect samples from 42 plots. The leaves collected from each species in each plot were combined (leaves of most species were small), weighed and scanned for area, using a flat bed scanner (Epson perfection V300) and image analysis software (ImageJ, [55]). Leaf samples were then dried in an oven for 72 hours at 60°C and re-weighed.

These leaf samples were then bulk sampled by species and treatment and analysed for total nitrogen and carbon concentration using a LECO CNS 2000 combustion analyser set at 1100°C. Total leaf phosphorus concentration was measured using a Varian Vista Pro ICPOES on samples digested in 5∶1 nitric∶perchloric acid (six samples were analysed per species per treatment) [56]. We measured leaf nitrogen and phosphorus concentration because extensive research has shown a stronger relationship between these nutrients within the leaf economic spectrum than other nutrients [57]. Six soil samples (core radius of 5 cm, 10 cm deep) were collected from each plot at the same time as the botanical surveys. Available soil nitrate, ammonium and phosphorus were analysed with colorimetric methods using a SEAL AQ2 [58]. Soil and leaf nutrient analyses were conducted by the Analytical Services Unit, School of Land and Food Sciences, the University of Queensland.

In September 2010, we measured assimilation rates (*A*_max_) of eight individuals of lovegrass and purple wiregrass within four fertilised plots and four unfertilized plots in each of the four blocks. We were very careful with our leaf selection, choosing young, intact leaves with a healthy appearance and growing in full sun. We took measurements between 6:00 am and 10:00 am over five days to standardize measurements. Because of the smaller size of woodlands lovegrass leaves and its low abundance, we were only able to measure four individuals within each of the treatments. We used a LI-COR LI-6400 photosynthesis system and the narrow leaf chamber LI-COR LI-6400-11. For assimilation rates ambient CO_2_ conditions were maintained at 400 µmol L^−1^, relative humidity at 40–50%, leaf temperature at 22–23°C and PAR at 1300–1530 µL L^−1^. To fill the chamber, multiple leaves growing in full sun were selected from each individual and leaf area was measured with a LI-COR, LI-3000c Portable Area Meter. We calculated photosynthetic nitrogen use efficiency (PNUE) as the ratio of *Amax* to leaf nitrogen.

### Data analysis

To analyse the effects of different treatments on species abundance and leaf traits, we developed Linear Mixed Effects Models (hereafter LMEM), using R 2.12.1 (R Foundation for Statistical Computing©) and the nlme package. We modelled the abundance (arc-sine transformed) of each study species in time 3 as a function of grazing and fertilizer treatments and abundance at time 0 as a covariate with a nested random effects structure of block/plot. We also modelled each of the leaf traits and soil nutrient levels as a function of the grazing and fertilizer treatments with a nested random effects structure of block/plot. Maximum likelihood was used when comparing nested models to simplify the model for fixed effects [59], [60]. We used diagnostic plots to check model assumptions [59]; there was no evidence of correlation of observations within groups and we assumed that within group errors were normally distributed. Finally, we used ANOVAs to assess the significance of the fixed effects within the LMEMs (Pinheiro and Bates, 2000). To analyse leaf nutrient concentrations, we used ANOVAs as opposed to LMEMs because these values were measured from bulked samples.

## Supporting Information

Table S1 Effect of treatments on Soil NO_3_, NH_4_ and PO_4_ levels in year 4. Results of an ANOVA conducted to assess the significance of the fixed effects for LMEMs of soil nutrient levels, with a fixed effects structure of grazing and fertilizer treatments, and a random effects structure of block/plot.
(DOCX)

Table S2 Effect of treatments on leaf nitrogen concentration, leaf carbon to nitrogen ratio and total leaf phosphorus concentration. Results of an ANOVA conducted for each of the three grass species.
(DOCX)

Figure S1 Soil nitrate, ammonium and phosphate levels taken across the treatments in year 3 of the field trial. e = grazing exclusion treatment, e+f = grazing exclusion and fertilized treatment, g = grazing treatment, and g+f = grazing and fertilized treatment.
(TIF)

Figure S2 Mean assimilation rates (± SE) for each species depending on the grazing and fertilizer treatments. **C** indicates the control treatment grazing/no fertilizer.
(TIF)
