# Supplementary material for: Plastic Traits of an Exotic Grass Contribute to Its Abundance but Are Not Always Favourable
Source: PLoS One. 2012 Apr 20;7(4):e35870. doi: 10.1371/journal.pone.0035870 (PMC3335023; doi:10.1371/journal.pone.0035870)
Supplement: Table S1 — Effect of treatments on Soil NO3, NH4 and PO4 levels in year 4. Results of an ANOVA conducted to assess the significance of the fixed effects for LMEMs of soil nutrient levels, with a fixed effects structure of grazing and fertilizer treatments, and a random effects structure of block/plot. (DOCX) [file pone.0035870.s001.docx]

Table S1: Effect of treatments on Soil NO_3_, NH_4_ and PO_4_ levels in year 4. Results of an ANOVA conducted to assess the significance of the fixed effects for LMEMs of soil nutrient levels, with a fixed effects structure of grazing and fertilizer treatments, and a random effects structure of block/plot.

| Parameter | Fixed effect | F-values (df as subscript), *P* value |
| --- | --- | --- |
| a) NO_3_ (mg/kg) | grazing treatment | *F*_1,2_ = 1.74, *P*<0.35 |
|  | fertilizer treatment | *F*_1, 58_ = 3.26, *P*<0.08 |
|  | grazing x fertilizer treatment | *F*_1, 58_ = 3.00, *P*<0.09 |
| b) NH_4_ (mg/kg) | grazing treatment | *F*_1, 2_ = 0.38, *P*<0.60 |
|  | fertilizer treatment | *F*_1, 58_ = 0.96, *P*<0.30 |
|  | grazing x fertilizer treatment | *F*_1, 58_ = 2.24, *P*<0.15 |
| c) PO_4_ | grazing treatment | *F*_1, 2_ = 30.53, *P*<0.03 |
|  | fertilizer treatment | *F*_1, 58_ = 11.67, *P*<0.001 |
|  | grazing x fertilizer treatment | *F*_1, 58_ = 1.11, *P*<0.30 |
