# Supplementary material for: Plastic Traits of an Exotic Grass Contribute to Its Abundance but Are Not Always Favourable
Source: PLoS One. 2012 Apr 20;7(4):e35870. doi: 10.1371/journal.pone.0035870 (PMC3335023; doi:10.1371/journal.pone.0035870)
Supplement: Table S2 — Effect of treatments on leaf nitrogen concentration, leaf carbon to nitrogen ratio and total leaf phosphorus concentration. Results of an ANOVA conducted for each of the three grass species. (DOCX) [file pone.0035870.s002.docx]

Table S2: Effect of treatments on leaf nitrogen concentration, leaf carbon to nitrogen ratio and total leaf phosphorus concentration. Results of an ANOVA conducted for each of the three grass species.

| Species/parameter | Fixed effect | F-values (df as subscript), *P* value |
| --- | --- | --- |
| a) Lovegrass: Total Nitrogen (% weight) | grazing treatment | *F*_1_ = 0.29, *P*<0.60 |
|  | **fertilizer treatment** | ***F*_1_ = 4.67, *P*<0.06** |
|  | grazing x fertilizer treatment | *F*_1_ = 1.17, *P*<0.35 |
| Total phosphorus (% weight) | **grazing treatment** | ***F*_1_ = 6.20, *P*<0.03** |
|  | fertilizer treatment | *F*_1_ = 1.40, *P*<0.30 |
|  | grazing x fertilizer treatment | *F*_1_ = 0.29, *P*<0.60 |
| C:N ratio | grazing treatment | *F*_1_ = 0.20, *P*<0.70 |
|  | **fertilizer treatment** | ***F*_1_ = 7.3, *P*<0.02** |
|  | grazing x fertilizer treatment | *F*_1_ = 0.50, *P*<0.50 |
| b) Purple wiregrass: Total Nitrogen (% weight) | **grazing treatment** | ***F*_1_ = 5.33, *P*<0.08** |
|  | fertilizer treatment | *F*_1_ = 1.33, *P*<0.30 |
|  | grazing x fertilizer treatment | *F*_1_ = 0.120, *P*<1.0 |
| Total phosphorus (% weight) | grazing treatment | *F*_1_ = 2.0, *P*<0.20 |
|  | fertilizer treatment | *F*_1_ = 0.50, *P*<1.0 |
|  | grazing x fertilizer treatment | *F*_1_ = 0.90, *P*<0.40 |
| C:N ratio | **grazing treatment** | ***F*_1_ = 7.17, *P*<0.06** |
|  | fertilizer treatment | *F*_1_ = 0.90, *P*<0.40 |
|  | grazing x fertilizer treatment | *F*_1_ = 0.01, *P*<0.90 |
| c) Woodlands lovegrass: Total Nitrogen (% weight) | grazing treatment | *F*_1_ = 2.8, *P*<0.14 |
|  | fertilizer treatment | *F*_1_ = 0.05, *P*<1.5 |
|  | grazing x fertilizer treatment | *F*_1_ = 0.80, *P*<0.85 |
| Total phosphorus (% weight) | **grazing treatment** | ***F*_1_ = 7.82, *P*<0.06** |
|  | fertilizer treatment | *F*_1_ = 3.56, *P*<0.10 |
|  | grazing x fertilizer treatment | *F*_1_ = 0.18, *P*<0.43 |
| C:N ratio | grazing treatment | *F*_1_ = 9.33, *P*<0.02 |
|  | fertilizer treatment | *F*_1_ = 0.14, *P*<0.26 |
|  | grazing x fertilizer treatment | *F*_1_ = 0.08, *P*<1.0 |
