# Supplementary figures and images for: Plastic Traits of an Exotic Grass Contribute to Its Abundance but Are Not Always Favourable
Source: PLoS One. 2012 Apr 20;7(4):e35870. doi: 10.1371/journal.pone.0035870 (PMC3335023; doi:10.1371/journal.pone.0035870)

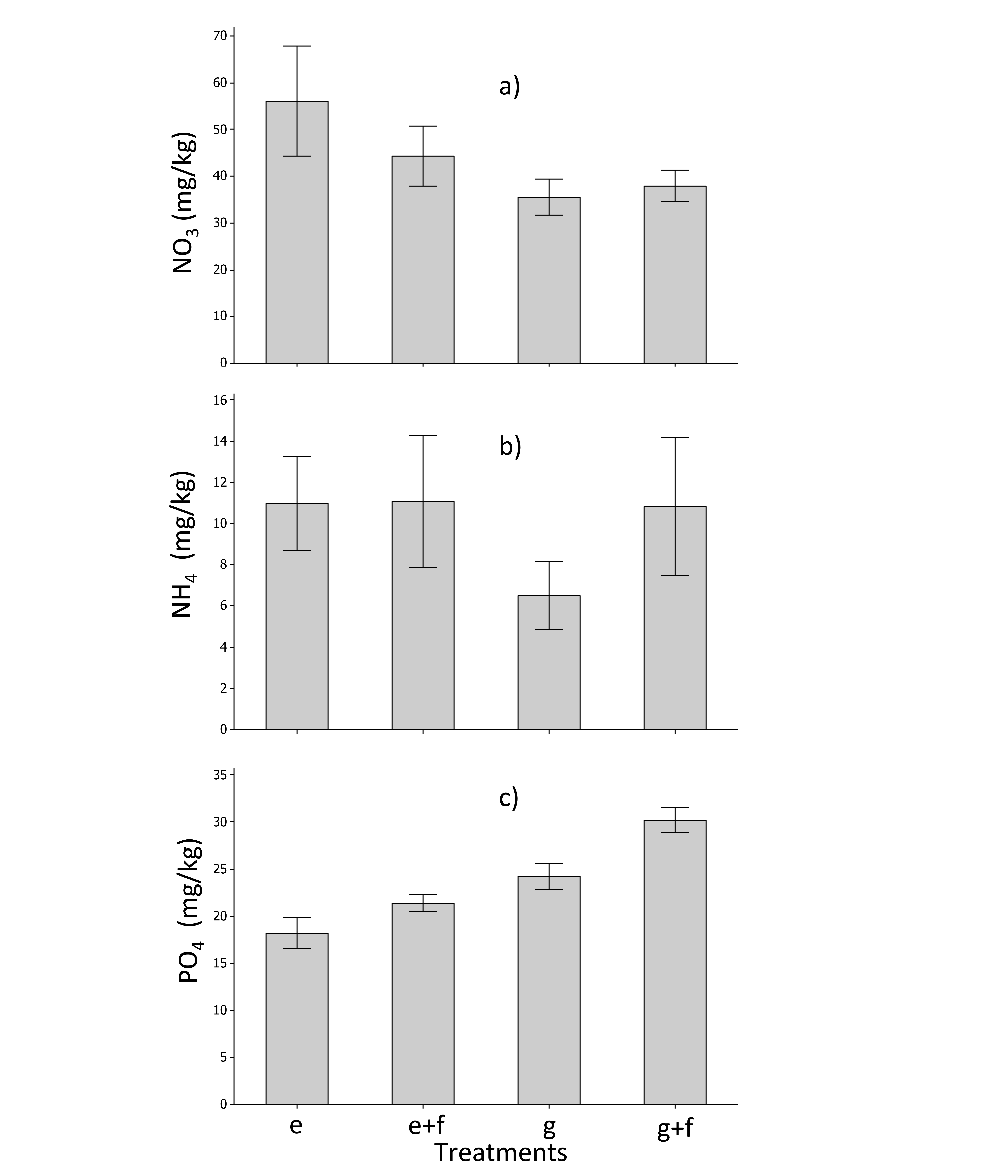

Supplement: Figure S1 — Soil nitrate, ammonium and phosphate levels taken across the treatments in year 3 of the field trial. e = grazing exclusion treatment, e+f = grazing exclusion and fertilized treatment, g = grazing treatment, and g+f = grazing and fertilized treatment. (TIF) [file pone.0035870.s003.tif]

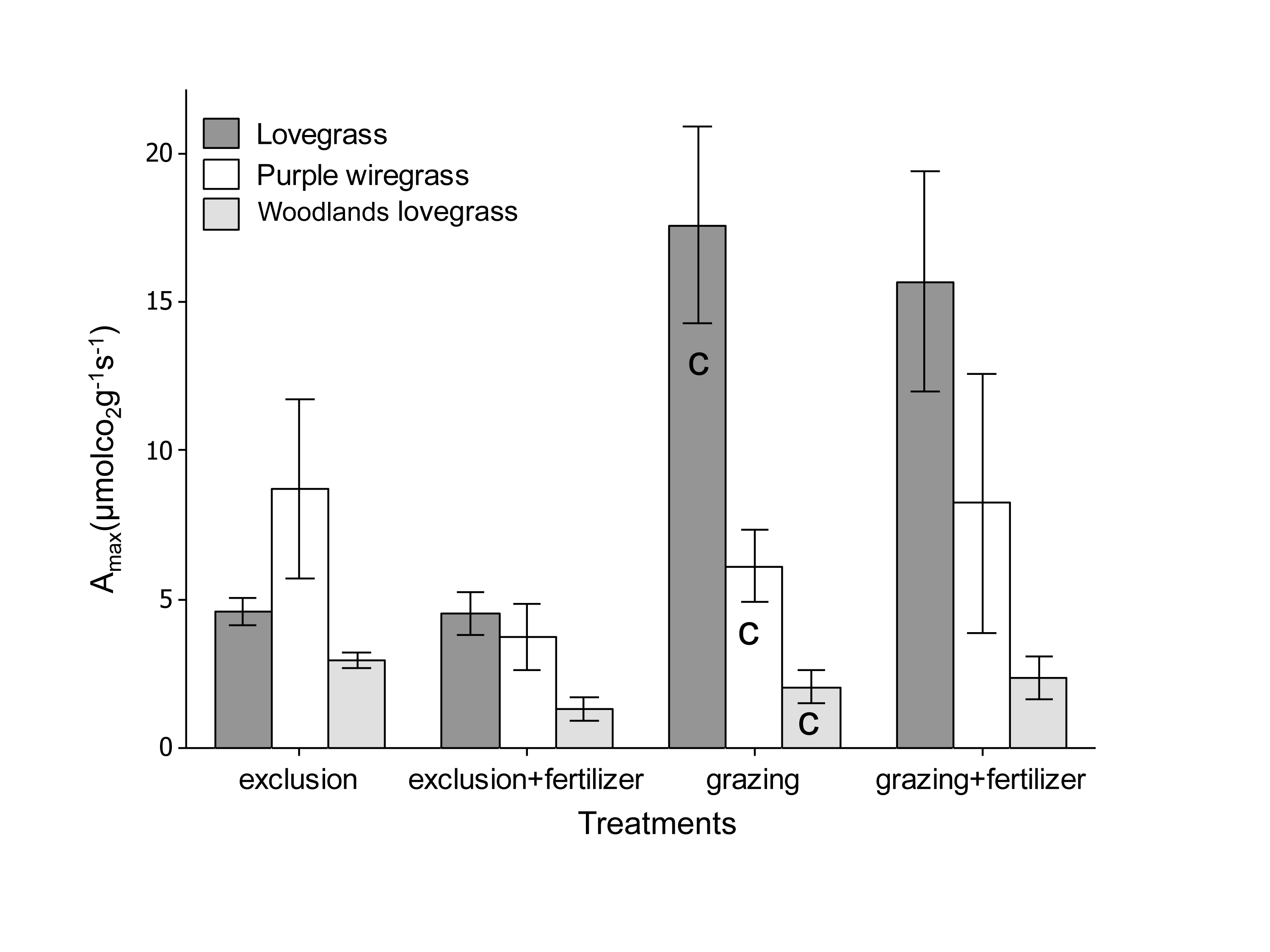

Supplement: Figure S2 — Mean assimilation rates (± SE) for each species depending on the grazing and fertilizer treatments. C indicates the control treatment grazing/no fertilizer. (TIF) [file pone.0035870.s004.tif]
